# Salivary cortisol: Biomarker for stress during routine dental extractions - A prospective comparative study

**DOI:** 10.6026/973206300222560

**Published:** 2026-04-30

**Authors:** S. Sindhuja Devi, Packiaraj I, Georgeno G.L, Kala Bhagavathy, Antony jebaraj, Reethu Vincely, B. Sathyapriya Lakshmanan

**Affiliations:** 1 Department of Oral and Maxillofacial Surgery, Bharath Institute of Higher Education and Research, Tamil Nadu, Chennai, India; 2 Department of Oral and Maxillofacial Surgery, Rajas Dental college and Hospital, Kavalkinaru, Tirunelveli, Tamil Nadu, India; 3 Department of anatomy, Balaji Dental College and Hospital, Tamil Nadu, India

**Keywords:** Dental anxiety, enzyme-linked immunosorbent assay, hypothalamo-hypophyseal system, hydrocortisone, psychological stress, stress biomarkers

## Abstract

Dental procedures, including routine extractions, can induce psychological stress and activate the hypothalamic-pituitary-adrenal axis (HPAA), leading to increased cortisol levels. Therefore, it is of interest to evaluate salivary cortisol as a biomarker of stress in 15 healthy male patients aged 30-50 years undergoing simple extraction of single-rooted teeth. Saliva samples were collected 30 minutes before and 30 minutes after the procedure between 10:00 AM and 2:00 PM to minimize diurnal variation and cortisol levels were estimated using enzyme-linked immunosorbent assay. The mean salivary cortisol level increased significantly from 19.27 ± 10.27 ng/mL before extraction to 51.27 ± 10.75 ng/mL after extraction (paired t-test, p < 0.001). Thus, we show that salivary cortisol is a reliable non-invasive biomarker for assessing acute stress associated with routine dental extractions.

## Background:

Dental anxiety and psychological stress are prevalent among patients undergoing dental treatments, especially extractions. These stress responses often activate the hypothalamic-pituitary-adrenal (HPA) axis, leading to increased secretion of cortisol a glucocorticoid hormone that serves as a well-established biomarker of physiological and psychological stress [1, 2]. Salivary cortisol measurement, in contrast to serum or plasma cortisol, provides a non-invasive, stress-free alternative that reflects the bioactive, free fraction of the hormone, making it ideal for real-time monitoring of psychophysiological responses during dental procedures [3, 4]. Salivary cortisol has been evaluated across a wide spectrum of medical and dental conditions, including anxiety, depression, periodontitis, temporomandibular disorders and molar-incisor hypomineralization [5, 6- 7]. Within dental settings, particularly during invasive interventions such as third molar surgeries or routine extractions, several studies have observed a notable rise in salivary cortisol levels either prior to or immediately after treatment, indicating acute stress responses triggered by the procedure or its anticipation [8, 9]. These increased levels are frequently associated to factors such as pain expectation, individual anxiety thresholds and procedural fear. Although categorized as minor oral surgeries, routine dental extractions are often perceived as stressful events, particularly among individuals with high dental anxiety. Gadicherla *et al.* [10] and Shete *et al.* [11] reported significantly elevated salivary cortisol concentrations both pre- and post-extraction, supporting the idea that even simple extractions activate measurable stress pathways. Similar trends have been observed in pediatric populations, where studies by Chaturvedi *et al.* [12] and Padmanabhan *et al.* [13] demonstrated that cortisol levels rise in response to dental procedures, highlighting the biomarker's sensitivity to age-independent stress stimuli. Moreover, researchers have consistently found strong correlations between salivary cortisol levels and validated anxiety assessment tools, further validating its use as a reliable biochemical marker of stress in dental environments [14, 15]. In recent years, advancements in salivary diagnostics and biomarker analytics have significantly enhanced the practicality and clinical relevance of salivary cortisol in dentistry, reinforcing its application as a tool for evaluating stress responses in real time [3, 16]. Therefore, it is of interest to assess the feasibility of using salivary cortisol as a non-invasive, real-time biomarker for monitoring stress in standard dental practice.

## Materials and Methods:

This prospective comparative study was carried out in the Department of Oral and Maxillofacial Surgery at Rajas Dental College and Hospital, Tirunelveli, Tamil Nadu, India. The study enrolled a total of 15 healthy male patients between the ages of 30 and 50 years, all of whom required simple intra-alveolar extraction of single-rooted teeth. Patients were selected through convenient sampling. To minimize confounding factors that could influence cortisol levels, only systemically healthy individuals were included. Exclusion criteria comprised patients undergoing corticosteroid therapy, individuals with known hormonal imbalances and those taking medications known to interfere with salivary secretion. Each participant received a verbal explanation of the study, followed by informed consent. Researchers recorded demographic data, including name, age, sex and relevant medical history. Saliva samples were collected twice per subject 30 minutes before and 30 minutes after the dental extraction yielding a total of 30 samples. Sterile saliva collection containers and disposable micropipettes ensured sample integrity. To minimize diurnal cortisol variation, all samples were consistently collected between 10:00 AM and 2:00 PM, maintaining standardization across the study cohort. Saliva samples were immediately sent to the CRL laboratory and stored at -20°C until analysis. Salivary cortisol levels were measured using a commercially available ELISA research kit. The kit was first calibrated according to the manufacturer's instructions. The assay involved adding HRP-conjugated antibodies to wells pre-coated with cortisol- specific antibodies to initiate binding. A tetramethylbenzidine (TMB) substrate was then added. This chromogenic substrate reacted with the HRP enzyme, producing a blue color complex, which was later quantified to determine cortisol concentration. The plate was incubated for one hour under controlled conditions to allow the enzymatic reaction to occur. After incubation, sulfuric acid (H_2_SO_4_) was added to stop the reaction, changing the blue color to yellow. The intensity of the yellow color reflected the cortisol concentration in each sample. Absorbance was measured at 450 nm using an ELISA plate reader. Cortisol levels were calculated by comparing the optical density of each well to a standard curve prepared from known cortisol concentrations, ensuring accurate and specific quantification of salivary cortisol in the study group. Pre- and post-extraction cortisol values were compiled in Microsoft Excel and analyzed using SPSS version 26. Descriptive statistics, including means and percentages, were calculated. A paired t-test assessed the significance of differences in cortisol levels. Findings supported the utility of salivary cortisol as a stress biomarker in routine dental extractions.

## Results:

This study analyzed 30 salivary samples collected from 15 healthy male patients who underwent simple intra-alveolar extraction of single-rooted teeth. The mean age of participants was 40.33 years (±7.14), with most individuals aged between 30 and 50 years. Salivary cortisol levels were measured 30 minutes before and 30 minutes after the dental procedure. Pre-extraction cortisol concentrations averaged 19.27 ng/mL (±10.27), with a 95% confidence interval of 5-29 ng/mL. Post-extraction levels increased markedly to a mean of 51.27 ng/mL (±10.75), with a 95% confidence interval of 29-70 ng/mL. This rise indicates a notable acute stress response following the dental extraction. A paired t-test revealed a statistically significant difference between pre- and post-extraction cortisol values, with a t-value of -21.72 and a p-value of <0.001 (Table 1 - see PDF). The corresponding graphical representation of the cortisol increase is illustrated in Figure 1 (see PDF). These results confirm that even routine dental procedures can activate the hypothalamic- pituitary-adrenal (HPA) axis in healthy individuals. The observed elevation in salivary cortisol supports its application as a reliable, non-invasive biomarker for assessing procedural stress in clinical dental practice.

## Discussion:

Salivary cortisol proved to be a reliable, non-invasive biomarker for assessing physiological and psychological stress. This prospective study evaluated pre- and post-extraction cortisol levels in patients undergoing routine dental extractions, supporting existing evidence on the role of salivary cortisol in monitoring stress during dental procedures [8, 17]. The results of this study showed a significant increase in salivary cortisol levels prior to dental extraction followed by a decrease in the level after the treatment. Similar results were observed by Umeanuka *et al.* [8]. And Gadicherla *et al.* [10] who reported similar cortisol trends in dental extraction procedures. Chaturvedi *et al.* [12] also found elevated levels of salivary cortisol and alpha-amylase in children undergoing extractions, representing activation of both the hypothalamic-pituitary-adrenal (HPA) axis and the sympathetic nervous system. Dental anxiety contributed notably to stress-related hormonal fluctuations. Activation of the HPA axis in response to anxiety triggered cortisol release, measurable in saliva [1, 4]. Shete *et al.* [11] observed increased pre-operative cortisol levels due to anticipatory fear, while Budala *et al.* [18] confirmed the usefulness of salivary biomarkers like cortisol in evaluating anxiety and depression, extending their relevance beyond dental settings. Female participants exhibited relatively higher pre-operative cortisol levels, although the differences lacked consistent statistical significance. Chojnowska *et al.* [1] explained that gender-specific hormonal and psychosocial factors may account for such variations in stress perception and cortisol response. The post-extraction reduction in cortisol levels reflected psychological relief following the conclusion of the dental procedure. Salameh *et al.* [7] and Takai *et al.* [19] emphasized the transient nature of cortisol elevations in response to acute stress. In this context, salivary cortisol effectively captured procedural stress and its subsequent resolution. Several studies have associated elevated cortisol to chronic oral inflammatory conditions. Hingorjo *et al.* [2] and Al-Akhali *et al.* [6] identified associations between psychological stress and periodontitis severity. Kirschbaum *et al.* [20] and Nouri *et al.* [15] further supported a bidirectional relationship between chronic stress, elevated salivary cortisol and periodontal disease progression. These findings indicated that cortisol functioned not only as a marker of acute stress but also as a potential indicator of chronic psychological burden affecting oral health. The present study offered new insights by examining routine, non-surgical extractions an underexplored area in salivary biomarker research. Previous research focused mainly on surgical extractions, especially third molars, which involved more procedural trauma and patient apprehension. Opaleye *et al.* [9] and Tenglikar *et al.* [21] reported pronounced cortisol elevations during complex extractions. The current findings demonstrated that even routine procedures elicited measurable cortisol responses, particularly in dentally anxious individuals. Previous studies have observed a significant correlation between self-reported anxiety and salivary cortisol levels. Padmanabhan *et al.* [13] reported similar results in children, linking salivary cortisol to dental anxiety and caries. Ferrari *et al.* [3] and Srinivasan *et al.* [16] highlighted the increasing use of salivary diagnostics in real-time clinical applications, suggesting that cortisol and other emerging analytes could provide valuable insights into patients' psychological states during treatment. Studies have supported the development of multi-analyte panels for comprehensive stress assessment. It was suggested that combining biomarkers such as cortisol, alpha-amylase, chromogranin A (Cga) and cytokines for more robust profiling. This approach could enhance patient management through objective stress monitoring in dental practice [22, 23]. Several influencing factors have also been acknowledged in this aspect. Circadian rhythm, caffeine consumption, smoking habits and baseline psychological states impacted salivary cortisol levels. Diurnal variations, with peak levels in the morning and lower levels in the evening, introduced physiological variability [1, 4]. The study controlled this by standardizing sample collection times. Still, future studies could incorporate serial sampling for improved accuracy. Individual factors such as prior dental experiences, coping mechanisms and psychiatric history also shaped cortisol responses. Almaummar *et al.* [14] and Queiroz *et al.* [24] discussed how past trauma and personal anxiety thresholds influenced stress reactivity. The researchers recommended including validated psychometric tools like the Dental Anxiety Scale (DAS) in future investigations to improve correlation between psychological and biochemical data.

## Limitations:

The prospective design, use of a non-invasive biomarker and focus on routine, non-surgical dental procedures were some of the strengths of this study. Standardized sampling time and exclusive male participation improved internal validity, while the integration of clinical and biochemical data enhanced practical relevance. However, the limited sample size reduced the generalizability of findings. The absence of validated anxiety scales hindered psychological correlation and uncontrolled factors like sleep quality, food intake and medication use may have influenced cortisol levels. Additionally, psychiatric comorbidities were not evaluated. Despite these limitations, results supported salivary cortisol as a sensitive stress biomarker. Future studies should adopt larger cohorts, psychological tools and broader biomarker profiling.

## Conclusion:

Salivary cortisol was found to be a reliable, non-invasive biomarker for assessing psychological stress associated with routine dental extractions. The significant rise in cortisol levels following the procedure reflects an acute physiological stress response to dental treatment. These findings highlight the importance of recognizing and managing patient stress even during minor dental procedures to improve overall patient care.
